# Clinical and Brain Imaging Findings in a Child with Vitamin B12 Deficiency

**DOI:** 10.3390/pediatric13040069

**Published:** 2021-10-25

**Authors:** Paola Feraco, Francesca Incandela, Roberto Franceschi, Cesare Gagliardo, Maria Bellizzi

**Affiliations:** 1 Neuroradiology Unit, Santa Chiara Hospital of Trento, 38122 Trento, Italy; 2 Section of Radiological Sciences, Department of Biomedicine, Neurosciences and Advanced Diagnostics, University of Palermo, 90127 Palermo, Italy; incandelaf.radiologia@gmail.com (F.I.); cesare.gagliardo@unipa.it (C.G.); 3 Pediatrics Unit, Santa Chiara Hospital of Trento, 38122 Trento, Italy; roberto.franceschi@apss.tn.it (R.F.); maria.bellizzi@apss.tn.it (M.B.)

**Keywords:** vitamin B12, magnetic resonance imaging (MRI), brain atrophy

## Abstract

Vitamin B12 (Vit-B12) deficiency is a rare and treatable cause of failure to thrive and delayed development in infants who are exclusively breastfed. Apart from genetic causes, it can be related to a malabsorption syndrome or when the mother follows a strict vegetarian or vegan diet, causing a low hepatic storage of Vit-B12 in the infant at birth. As the neurological symptoms are nonspecific, a brain magnetic resonance imaging (MRI) exam is usually performed to rule out primary causes of neurodevelopmental delay. Findings related to brain atrophy are usually observed. A favorable response is achieved with Vit-B12 therapy, and neurological symptoms dramatically improve within a few days after the treatment. We present the case of an infant with severe Vit-B12 deficiency, exclusively breastfed by his young vegan mother, and whose clinical symptoms together with MRI findings improved after treatment. Brain atrophy recovery after Vit-B12 therapy has been seldom documented.

## 1. Introduction

In developed countries, vitamin-B12 (Vit-B12) deficiency is a rare and treatable cause of failure to thrive and delayed development in infants who are exclusively breastfed. It is usually the result of a malabsorption syndrome in the mother or when the mother follows a strict vegetarian or vegan diet, causing a low hepatic storage of Vit-B12 in the infant at birth. Moreover, a genetic etiology has also been documented [1,2]. In exclusively breastfed infants, Vit-B12 deficiency can cause serious clinical symptoms and neurological abnormalities that usually present around or after the fourth month of life [2,3]. These symptoms are nonspecific and include developmental delay, irritability, hypotonus, weakness, fatigue, and failure to thrive [1,3]. A brain MRI exam is usually performed to rule out primary causes of neurodevelopmental delay. Usually, together with macrocytic anaemia (which is related to Vit-B12 deficiency) and developmental psychomotor regression, findings related to brain atrophy might be observed, helping to direct the diagnosis [2,3,4]. A favorable response is achieved with Vit-B12 therapy, and neurological symptoms usually start to improve within a few days after the treatment [3,4,5]. We present the clinical history and describe the brain MRI findings of an infant with severe Vit-B12 deficiency who had been exclusively breastfed by his young vegan mother, and whose clinical symptoms together with MRI findings improved after treatment. To the best of our knowledge, brain atrophy recovery after Vit-B12 therapy has been seldom documented by MRI [6].

## 2. Case Presentation

A nine month old boy was referred to us for developmental regression. The infant, exclusively breastfed, was born at 37 weeks after labor was induced for growth retardation. At birth, he weighed 2130 g (<3rd centile) and had a birth length of 43.8 cm and a cranial circumference of 34 cm. At four months of age, his pediatrician noted a failure to thrive associated with psychomotor regression and hypotonia. He had normal strength and brisk reflexes, was able to follow objects visually, and smiled occasionally. No babbling was observed. The findings of the general physical examination were within the normal range without dysmorphic features. 

An observation was suggested. During the follow-up, at six months the patient began to lose appetite, vomiting, and refusing to wean. At nine months, his weight was 5885 g (<3rd percentile), he was pale, hypotonic, with no reaction to external stimuli. Psychomotor regression was confirmed.

The complete laboratory investigations are summarized in Table 1.

In particular, at nine months a severe megaloblastic anaemia (Hb 4.8 g/dL, MCV 100 fL) and leukopenia (WBC 3.500 × 109/L) were detected, and the serum Vit-B12 level was undetectable (normal range: 197–866 pg/mL). Vitamin B12 was tested with electrochemiluminescence (ECLIA), with a limit of detection of 100 pg/mL. Other tests have been performed to confirm vitamin B12 deficiency. In particular, the plasma homocysteine level result was markedly elevated (>50 µmol/L; normal: 5–15 µmol/L), and the organic acid urine level test showed an increased methylmalonic acid concentration (> 260 nmol/L).

**Figure 1:**
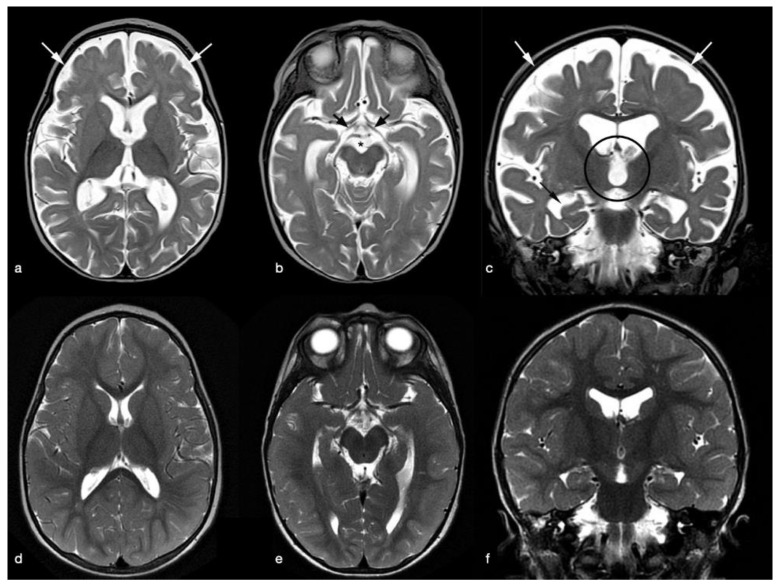
MRI exams performed (**a**–**c**) at admission to hospital and (**d**–**f**) 10 months after the start of Vitamin B12 treatment. (**a**,**b**) Axial (**d**,**e**) and coronal (**c**,**f**) T2-weighted images. There is evidence of a mild cerebral atrophy characterized by an enlargement of cortical sulci and subarachnoid spaces (white arrows in (**a**,**c**)). Enlargement of the lateral and third ventricles (open circle and black arrow in (**c**)), and the perimesenchephalic cistern (asterisk in (**b**)). Thinning of the post-chiasmatic optic nerves (black arrows in (**b**)). (**d**–**f**) Disappearance of all structural alterations after therapy.

**Figure 2:**
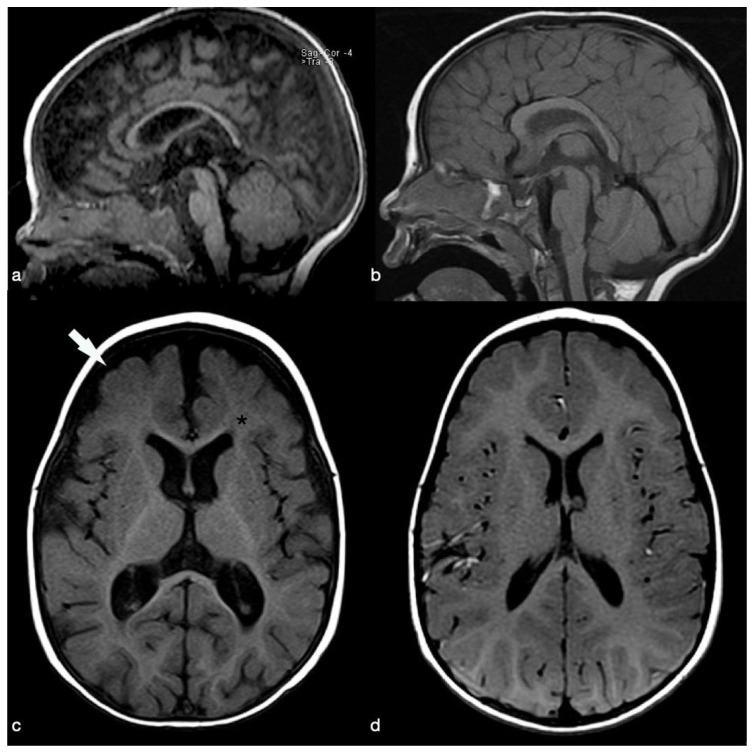
(**a**,**b**) Sagittal and (**c**,**d**) axial T1-weighted images of (**a**–**c**) the admission and follow-up MRI exams. Enlarged subarachnoid spaces (arrow in (**c**)) and lateral ventricles. Myelination is considered normal for the patient’s age (asterisk in (**c**)). (**b**,**d**) Substantial recovery of the structural changes after therapy, (**c**,**d**) an improvement of the white matter amount is evident in particular.

The electroencephalogram showed a diffuse slowing of basic rhythms. A conventional MRI (1.5 T GE Discovery MR450 scanner; 32-channel head coil;) obtained at nine months and performed to rule out primary causes of developmental delay revealed atrophic changes with a global prominence of the cerebral sulci and enlargement of the subarachnoid space and perimesencephalic cisterns and diffuse ventricular dilatation. Thinning of the optic nerves and corpus callosum was also detected. These findings were consistent with cerebral atrophy; myelination was considered appropriate for the age (Figure 1 and Figure 2).

Due to the presence of a low Vit-B12 blood level, the diagnosis was extended to rule out pernicious anaemia, and the gastric anti-parietal cell antibody (APCA) level was assessed. The result of this test was negative, as were the metabolic disease screening tests.

As the infant had been exclusively breastfed, his mother underwent biochemical and hematological tests. Although during pregnancy labor and breast-feeding she did not present any particular symptoms or signs related to Vit-B12 deficiency, the exams revealed that her serum Vit-B12 level was also undetectable and her gastric APCA test was consistent with pernicious anaemia. Moreover, it was only on this occasion that we discovered that she kept a strict vegan diet. 

To correct the child’s severe anaemia, red cell transfusions and parenteral Vit-B12 supplementation were administered. Intramuscular Vit-B12 administration was commenced after discharge (500 μg/week for five months, followed by 500 μg every other week, and then 1000 μg every month).

The child’s biochemical and hematological parameters had normalized by 15 days after admission (see Table 1). During his stay in hospital, he showed a prompt neurological improvement: he started smiling again and was no longer either lethargic or hypotonic, and he regained the psychomotor skills that had regressed. At 15 months, a significant clinical improvement in the boy’s conditions was observed: his weight was 8150 g, his Vit-B12 level was 736 pg/mL, and his hematological values were unremarkable. Further neurological improvement was observed, and a follow-up MRI scan showed the size of the subarachnoid spaces and cerebral ventricles to be normal, related to an improvement of the white matter amount, in keeping with a substantial recovery from the cerebral atrophy including in terms of the optic nerves morphology (see Figure 1 and Figure 2).

## 3. Discussion

The maternal Vit-B12 status is the major factor influencing the severity of a deficiency in the substance in breastfed infants. It may be caused by unrecognized pernicious anaemia, pre-existing malabsorption syndrome, and, nowadays, a strict vegan or vegetarian diet. Genetic Vit-B12 metabolism defects may also occur [4]. As humans do not synthesize Vit-B12, a diet lacking animal products (such as meat, liver, fish, eggs or milk) can lead to a deficiency in the substance [3,4,7]. Clinical symptoms of deficiency only present when the Vit-B12 body stores are severely depleted. Indeed, in adulthood, the average body stores compensate the daily losses, and a diet devoid of Vit-B12 does not usually induce clinical symptoms for many years. On the other hand, the hepatic store of a normal new-born can last eight months, but this period may be shorter if the mother has a Vit-B12 deficiency [8,9]. Moreover, if this physiological decrease is associated with a marginal hepatic storage and an inadequate supply, the infant’s development may be halted, and the related clinical symptoms may present, as in our case [9,10]. Indeed, although the patient’s birth weight was below the third centile for growth, his early development was normal and his symptoms started at six months of age. Failure to thrive and a decrease in the growth rate, including head circumference, may occur as reported above. Vit-B12 deficiency primarily affects the central nervous system (CNS) and hematopoiesis, but also tissues with a fast mitotic activity such as the epithelium of the digestive tract [3,11].

The relationship between Vit-B12 deficiency and neurological symptoms is poorly understood. Studies on children with inborn homocysteine re-methylation defects suggest that an abnormality in the methionine synthetase reaction, in which Vit-B12 is an important cofactor, is the main cause of the neurological effects of Vit-B12 deficiency [2]. Furthermore, propionyl-CoA accumulation, resulting from an unsuccessful Vit-B12-dependent reaction, leads to an odd chain fatty acid synthesis, resulting in the incorporation of large amounts of unusual fatty acids in the nerve sheaths, and ultimately in altered neural functions [7,8]. The acquisition of cognitive skills coincides with the CNS myelination pattern, which is most active in the first six months of life, and consequently myelination defects secondary to Vit-B12 deficiency can have significant effects in that they alter the function and conduction velocity in multiple regions of the brain, contributing to a delayed acquisition of cognitive skills. Moreover, the consequent brain atrophy leads to a regression of those skills already acquired [4,5,10]. As in our case, the most common symptoms include hypotonia and brisk reflexes, irritability or lethargy, and developmental delay and even regression. Epileptic seizures and movement disorders have been described [2,3,4,5,11,12]. However, as the neurological symptoms are nonspecific, the first-line diagnostic work-up usually also includes a brain MRI. Despite neurological Vit-B12 deficiency symptoms being widely described, only a few cases describing brain MRI features have been reported, with only one that demonstrated atrophy recovery after therapy [6,13,14].

Features consistent with cerebral atrophy include dilation of the ventricles and subarachnoid spaces. As in our case, optic nerve thinning is another hallmark of the disease [3,10]. Subdural effusions have also been described [4]. However, several neurogenetic and metabolic disorders, characterized by neurodevelopmental delay, also show nonspecific brain atrophy on the MRI, including mitochondrial disorders, hereditary spastic paraplegias and organic acid disorders [13].

Although a benign enlargement of the subarachnoid space and mild ventriculomegaly could explain that the brain MRI findings as well as follow-up changes could be related to the developmental process, we strongly believe that the imaging changes described were related to a profound Vit-B12 deficiency, which led to the appearance of neurological symptoms, as previously described [4,10,14].

On the other hand, the presence of megaloblastic anaemia together with neurological symptoms and brain atrophy should arouse a suspicion of Vit-B12 deficiency in infancy [14,15]. As in our case, treatment leads to progressive clinical improvement [2], also in terms of brain atrophy, as confirmed by the MRI follow-up exam. The mechanism underlying reversible brain atrophy is unknown, but in patients with Vit-B12 deficiency it could be directly proportional to the amount of Vit-B12 supplementation. An early diagnosis and initiating administration are essential.

However, although Vit-B12 supplementation leads to a rapid clinical and morphological improvement, there are concerns regarding the long-term prognosis, as the child may be left with long-term intellectual problems [2,3,7,8,16]. Hence, in order to avoid development regression and irreversible neurological damage in exclusively breastfed infants, Vit-B12 supplementation should be provided during pregnancy for strict vegetarian and vegan mothers and those with pernicious anaemia. MRI is an important instrument to rule out some other causes responsible for the symptoms, and the clinical assessment after therapy administration remains the golden rule to evaluate a patient’s response. However, MRI might be useful in monitoring brain changes in those cases without a clinical recovery, and further studies with a large patient sample are also needed to assess morpho-structural features related to Vit-B12 deficiency and their relationships with the long-term clinical outcome.

## Figures and Tables

**Table 1:** Principal abnormal laboratory findings at admission and their changes at discharge and at 10-months follow-up.

Parameters	Admission	Discharge	10-Months Follow-Up
WBC	3 × 10^9^/L (6–16) *	8.1 × 10^9^/L	8.2 × 10^9^/L
RBC	1.5 × 10^12^/L (4–5.1) *	3.9 × 10^12^/L	4.97 × 10^12^/L
HB	4.8 g/dL (10.3–13) *	11.4 g/dL	12.2 g/dL
HTC	15% (30 × 38.5) *	37.2%	36.2%
MCV	100 fL (70–90) *	93 fL	72.8
MCH	32 pg (23–29) *	28 pg	23.9 pg
RDW	25.4% (<15) *	18%	15.7%
Reticulocytes	16 × 10^9^/L (27–99) *	132 × 10^9^/L	87 × 10^9^/L
Vitamin B12	<100 pg/mL (197–866) *	630 pg/mL	736 pg/mL
Bilirubin, Total	1.4 mg/dL (0–0.5) *	0.4 mg/dL	0.3 mg/dL
Bilirubin, Direct	0.3 mg/dl (0–0.3)	0.1 mg/dL	0.1 mg/dL
LDH	1602 U/L (0–470) *	1049 U/L	550 U/L
Haptoglobin	<0.078 g/L (0–2.27)	0.24 g/L	0.5 g/L

WBC, white blood cells; RBC, red blood cells; Hb, haemoglobin; Htc, Haematocrit; MCV, Mean corpuscular volume; MCH, Mean cell haemoglobin; RDW, Red Blood Cell Distribution Width; LDH, lactate dehydrogenase; * out of range.
